# Synthetic tissues

**DOI:** 10.1042/ETLS20190120

**Published:** 2019-10-18

**Authors:** Hagan Bayley, Idil Cazimoglu, Charlotte E.G. Hoskin

**Affiliations:** Department of Chemistry, University of Oxford, Oxford, U.K.

**Keywords:** 3D printing, artificial, droplets, synthetic biology, synthetic cells, tissues

## Abstract

While significant advances have been achieved with non-living synthetic cells built from the bottom-up, less progress has been made with the fabrication of synthetic tissues built from such cells. Synthetic tissues comprise patterned three-dimensional (3D) collections of communicating compartments. They can include both biological and synthetic parts and may incorporate features that do more than merely mimic nature. 3D-printed materials based on droplet-interface bilayers are the basis of the most advanced synthetic tissues and are being developed for several applications, including the controlled release of therapeutic agents and the repair of damaged organs. Current goals include the ability to manipulate synthetic tissues by remote signaling and the formation of hybrid structures with fabricated or natural living tissues.

## Introduction

In this Perspective, synthetic tissues are defined as organized three-dimensional (3D) collections of non-living communicating synthetic compartments or cells. They are built from the bottom-up with biological or synthetic parts, and can, therefore, be inexpensive and easy to handle. While their simple nature may impart a lack of sophistication, it does mean that their properties are predictable. Furthermore, because their constituents can include components not found in natural tissues, there are no rules constraining their fabrication. Given the intriguing potential applications, especially in medicine, research in the area of synthetic tissues is intensifying.

## Natural tissues and organs

**Figure 1.**
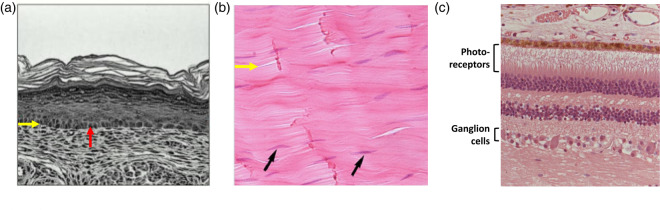
Arrangements of cells in living tissues. (**a**) Light micrograph of a section of the skin from the back paw of a mouse. Basement membrane (red arrow); basal epithelial cell (yellow arrow) [43] (Adapted with permission of Rockefeller University Press; permission conveyed through Copyright Clearance Center, Inc. Arrows added). (**b**) Light micrograph of a longitudinal section of human Achilles tendon. Collagen fiber bundles (yellow arrow); fibroblasts (black arrows) [44] (Adapted under CC BY 4.0. Image cropped, yellow arrow added). (**c**) Light micrograph of a section of the human retina. The cells are patterned to permit light detection and signal transmission to the brain [45] (Adapted under CC BY 3.0. Image cropped, labels added). Tissues of both high and low cell density are illustrated (‘**a**’ and ‘**b**’). Cells at high densities in tissues can be patterned (‘**c**’).

Natural tissues comprise collections of cells and are thereby compartmented [1]. The cells themselves contain sub-compartments such as endosomes and mitochondria. In some tissues, the neighboring cells are mostly in contact (e.g. skin, heart) and in other tissues, they reside within a high fraction of extracellular matrix (ECM) (e.g. cartilage, bone) [2,3] (Figure 1a,b). The cells interact with each other, allowing tissues to perform far beyond the sum of their parts. The interactions can be direct, mediated for example by gap junction proteins that span the two plasma membranes of neighboring cells, or indirect, mediated for example by diffusible effectors. The cells in natural tissues are often patterned in a manner that contributes to the functional properties of a tissue (Figure 1c). Important aspects of these properties have been replicated in synthetic tissues (see below). Other aspects, such as the ability of tissues to adapt to conditions and be repaired, which involve cell division, differentiation, and migration, may prove more difficult to emulate. Organs are found in higher animals and comprise specialized tissues supported by connective tissue, vascularization and the nervous system. The fabrication of synthetic organs is a distant goal, and the reliable production of synthetic tissues will be the first step in that direction.

## Synthetic tissues

Synthetic tissues are materials designed to substitute for natural tissues and even exhibit enhanced properties. They generally comprise a 3D-patterned collection of compartments (usually picoliters in volume) that can communicate with each other and with the environment. In the present context, synthetic tissues are built from the bottom-up and do not contain living cells. Indeed their design need not be restricted by attempts to strictly mimic nature.

**Figure 2.**
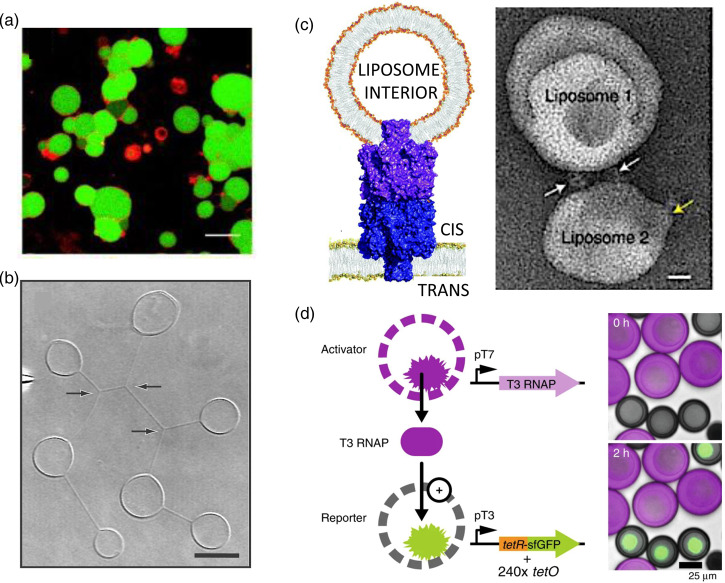
Synthetic tissues from assemblies of compartments. (**a**) ‘Colonies’ of giant lipid vesicles. Vesicles with negative surface charge were aggregated with poly-l-arginine [4] (Republished with permission from Wiley-VCH, Copyright 2012). Scale bar 30 µm. (**b**) Lipid vesicles connected by nanotube networks [46] (Republished with permission from the National Academy of Sciences, Copyright 2002). Arrows mark three-way junctions. Scale bar 5 µm. (**c**) An engineered dimeric α-hemolysin pore designed to act as a gap junction [8] (Labels reproduced). Left: cartoon (not to scale) showing the pore connecting a liposome and a planar lipid bilayer. Right: transmission electron micrograph showing the dimeric pore connecting two liposomes. (**d**) Diffusion-based communication between cell-mimics comprising DNA-containing hydrogel compartments within porous polymer membranes. A diffusive signaling molecule, here T3 RNA polymerase (T3 RNAP), effects the expression of a reporter gene in the recipient cell-mimic [10] (Republished under CC BY 4.0).

Attempts have been made to produce synthetic tissues from collections of lipid vesicles, each bounded by a single bilayer [4,5] (Figure 2a). In these systems, the compartments cannot communicate readily; although intervesicular transport through lipid nanotubes [6] (Figure 2b) and through pores in closely juxtaposed bilayers [7] have been demonstrated. Engineered membrane proteins that, like gap junctions, can span two bilayers are likely to prove more useful in this regard [8] (Figure 2c). Means to signal between dispersed vesicles and other containers with diffusible molecules have been demonstrated and might be extended to synthetic tissues [9–11]. For example, Niederholtmeyer et al. produced porous cell-mimics able to communicate with diffusive protein signals (Figure 2d) and thereby detect cell density [10].

**Figure 3.**
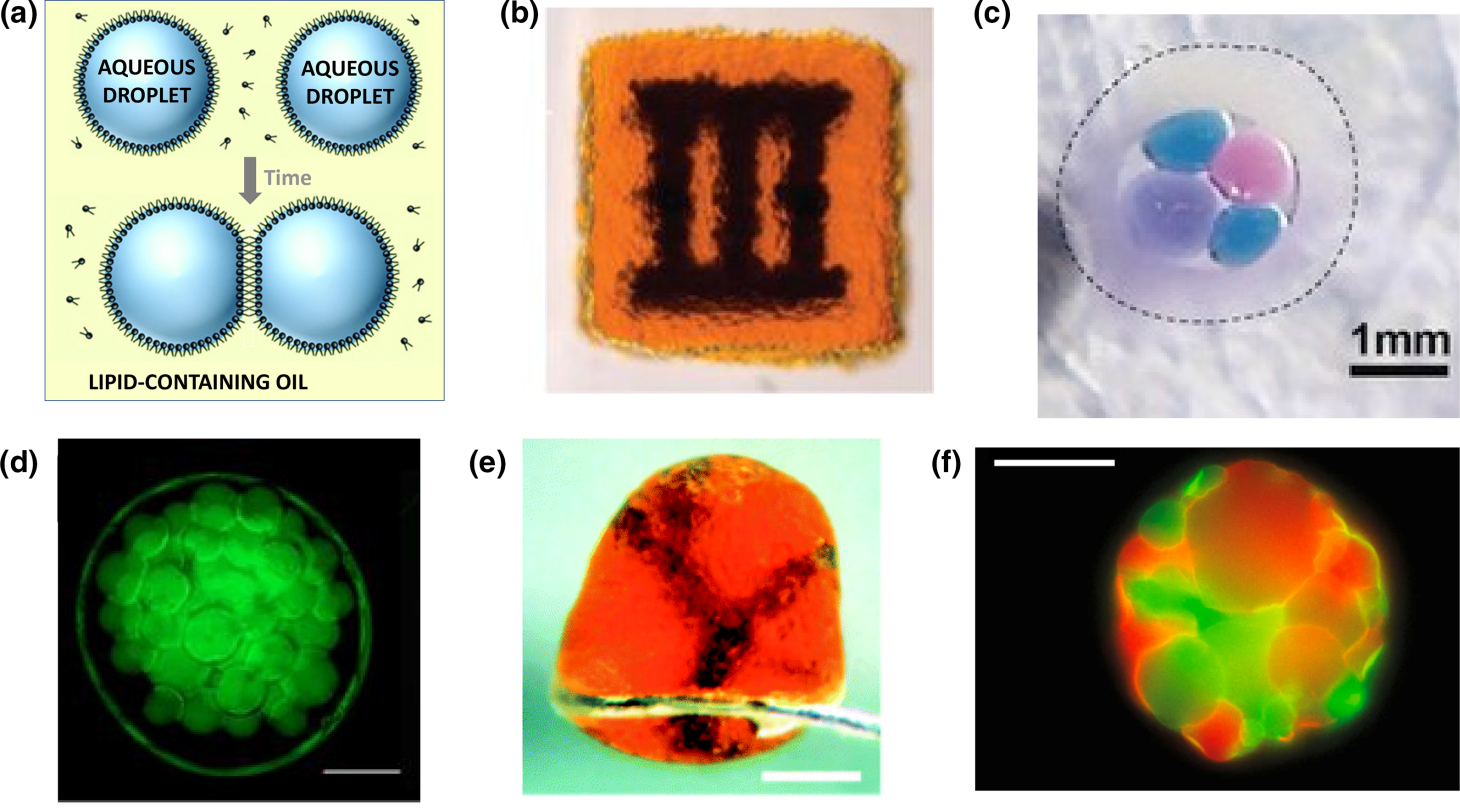
Synthetic tissues based on droplet-interface bilayers (DIBs). (**a**) Formation of a DIB in a lipid-containing oil [12] (Adapted with permission from The Royal Society of Chemistry. Labels added). (**b**) A 3D-printed patterned network based on DIBs [13] (Republished with permission from AAAS). (**c**) Droplets connected by DIBs encapsulated in oil sealed within an alginate shell obtained by a microfluidics approach [15] (Adapted under CC BY-NC 4.0. Image cropped). (**d**) An assembly of aqueous droplets formed by injection into an oil drop encased in agarose [14] (Republished under CC BY 4.0). Scale bar 1 mm. (**e**) A patterned network in an aqueous environment after 3D printing within a suspended oil drop [13] (Republished with permission from AAAS). Scale bar 400 µm. (**f**) Spheroidal assembly of a coacervate droplet: a protein-polymer ‘proteinosome’ [21] (Republished with permission from Springer Nature, Copyright 2018). Scale bar 50 µm.

Synthetic tissues built from picoliter droplets connected by interface bilayers (DIBs) [12] (Figure 3a) have reached a more sophisticated state of development. DIB-based synthetic tissues can be fabricated by 3D printing in a lipid-containing oil, which allows patterning of the compartments, which can be regarded as synthetic cells [13] (Figure 3b). Because the compartments are separated by individual, rather than double, bilayers, it is simpler to install communication between them with membrane proteins, including pores, channels, and transporters [12]. Oil drops containing clusters of droplets can be stabilized by encapsulation in hydrogels [14,15] (Figure 3c,d). After transfer to aqueous media, 3D droplet networks are bounded by lipid bilayers facilitating communication with the environment [13,16] (Figure 3e).

A third class of synthetic tissues might be assembled from synthetic cells comprising coacervate droplets. The investigation of membraneless organelles in living cells is an intriguing area of current research [17]. These organelles consist of functional components embedded within coacervate droplets formed by liquid–liquid phase separation. It has long been proposed that the first cells were membraneless droplets [18]. Accordingly, the investigation of interactions between these structures is an important endeavor [19–21] (Figure 3f).

Several challenging issues remain in the construction of synthetic tissues. These include the incorporation of functional sub-compartments (organelles), which has been achieved in synthetic cells (e.g. [22]), and the scaling up of production to achieve dimensions that will be useful for medical applications.

## Functionalized synthetic tissues

**Figure 4.**
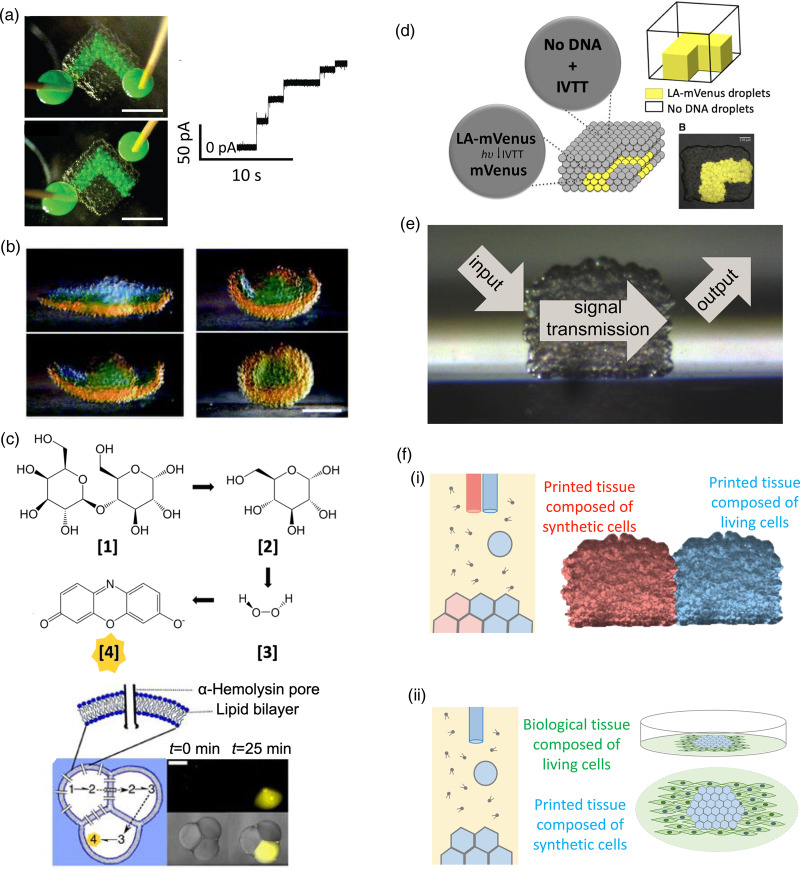
Functional synthetic tissues. (**a**) A conductive pathway in a 3D-printed synthetic tissue [13] (Republished with permission from AAAS. Axis labels reproduced). When electrodes are placed at both ends of the pathway (upper panel), a current flows through the system. Scale bar 500 µm. (**b**) A folding printed synthetic tissue based on osmotic water flow [13] (Republished with permission from AAAS). Scale bar 200 µm. (**c**) A three-droplet system, which performs a three-step enzymatic reaction cascade, one step in each droplet. The fluorescent resorufin product appears yellow [25] (Adapted under CC BY 4.0. Reaction schematic and labels reproduced). Scale bar 250 µm. (**d**) Light-activated expression of the fluorescent mVenus protein in a 3D-printed network [28] (Adapted under CC BY-NC 4.0. Labels reproduced). (**e**) Signaling systems in synthetic tissues. There is a range of inputs, modes of transmission and outputs (see the text). (**f**) Hybrid tissues: (i) synthetic tissue and droplets containing living cells printed together and (ii) synthetic tissue embedded in living tissue.

To illustrate the functional properties of synthetic tissues, we focus on DIB-based materials with which most advances have been made (Figure 4).

Transport between compartments in synthetic tissues has been mediated by using protein pores, notably α-hemolysin [23], which is a robust bacterial pore-forming protein that has proved useful in several areas of biotechnology. In small multi-compartment systems, the movement of Ca^2+^ ions can be monitored with fluorogenic dyes both between compartments and between compartments and the environment [16]. While the long-distance movement of Ca^2+^ has not been observed in this way, electrical signals have been sent through larger structures in the form of ionic currents. Rapid directional transmission can be achieved by patterning pathways containing the α-hemolysin pore by 3D printing [13] (Figure 4a).

Water can move rapidly through lipid bilayers in the absence of pores, and osmotic flow has been used to produce irreversible shape changes in synthetic tissues [13] (Figure 4b). Recently, reversible shape changes in systems built from a few droplets have been achieved by using temperature-responsive polymers (Downs and Bayley, unpublished observations) and this approach holds promise for controlling shape and movement in synthetic tissues.

Membrane proteins other than pores might be used to functionalize synthetic tissues and further work in the area is needed. Several additional proteins have been used in simple droplet systems [12], notably lactose permease, which was shown to transport a fluorescent sugar analog against a concentration gradient, driven by a pH gradient [24].

Enzymes have been incorporated into a simple three-droplet system [25] (Figure 4c) and their use would be an advantageous addition to synthetic tissues. Cell-free transcription and translation systems were first used to produce active proteins in droplet pairs [26], and this approach has been usefully extended to patterned synthetic tissues. Notably, a light-activated system, in which a transcriptional promoter is blocked by proteins that can be removed by photocleavage of a chemical linker, has been developed so that protein synthesis can be turned on at will with spatial control [27] (Figure 4d). For example, the synthesis of α-hemolysin in selected compartments of a synthetic tissue permitted the light-activated production of electrical signaling pathways [28].

Ultimately, the functions of synthetic tissues (such as protein synthesis) must be maintained by an energy source. Ionic gradients [29] and encapsulated ATP [26] can provide energy in the short-term. Long-term energy production will require an outside source, such as light to drive an ion pump (e.g. bacteriorhodpsin) [29]. Proton gradients have been converted to ATP in synthetic cells (e.g. [22]), and this approach is likely to be useful in synthetic tissues.

## Signaling in synthetic tissues

An important future goal for researchers on synthetic tissues is the production of more sophisticated signaling systems. Such systems will include receptors for physical and chemical inputs, means to process the signals and transmit them through the tissues, and finally various outputs (Figure 4e). Considerable progress has been made in this area with engineered living cells [30,31], providing useful lessons for signaling by synthetic systems. A wide variety of receptors are available, from proteins such as bacteriorhodopsin, which can act as a light receptor [32], to completely synthetic receptors, which can respond to various inputs [33,34]. The fast transmission of signals through synthetic tissues can be electrical, as described earlier, while promising alternatives, including mechanical transmission [35] and the propagation of chemical waves [36], remain to be full exploited. In an inventive approach, slow transmission by diffusion from a sender cell, either directly through lipid bilayers or through the α-hemolysin pore, has been used to produce a traveling wave of fluorescence mediated by a gene circuit [37]. Numerous potential outputs from synthetic tissues can be envisioned or are in development. They include the release of small molecules or biologicals, which might be ‘prepackaged’ (Booth and Bayley, unpublished observations) or made *in situ* by, for example, cell-free protein synthesis. Synthetic tissues that include responsive polymers might produce mechanical responses, such as gripping or useful shape changes or movement. Electrical outputs would include the patterned injection of ions into a biological interface. Simple computational processing whereby a synthetic tissue can integrate two or more incoming signals or choose between two or more outputs, also remains to be developed, although slow versions have been demonstrated in systems containing just a few droplets [38].

## Hybrid tissues

Living cells can also be assembled into patterned 3D structures by various processes [31,39,40] including 3D printing in droplets [41]. Similarly, living cells might be included in selected compartments within synthetic tissues. The ability to integrate these materials with tissues in a living animal is of considerable interest (Figure 4f). In one possibility, synthetic tissues designed to release therapeutic agents, perhaps in response to an external signal, might simply be implanted in a cavity without the need for full integration with neighboring tissues. In other cases, close integration might be required, which might be achieved, for example, by electrical coupling between the outermost bilayers of the two components [8]. Patterned electrical signals can be produced by droplet arrays [32] and printed versions might be used to control excitable tissues. The effects of weak electric fields on tissues and organs are an intriguing area of investigation that impacts development and tissue regeneration [42]. Synthetic tissues that generate patterned electrical signals could have a significant impact in this area. In all of these cases, the lifetimes of synthetic tissues *in vivo* and their immunogenicity will be of concern.

## Summary

- While much progress has been made on non-living synthetic cells, less work has gone into the fabrication of synthetic tissues
- Communicating compartments partitioned by DIBs are the basis of the most advanced synthetic tissues
- Synthetic tissues can incorporate features that go beyond mimicking nature
- Synthetic tissues are being developed for several applications including the controlled release of therapeutic agents and the repair of damaged organs
- Current goals include the powering of synthetic tissues with an energy source, the manipulation of synthetic tissues by remote signaling and the establishment of hybrid structures with tissues containing living cells

## Abbreviations

DIB: bilayer at the interface between aqueous droplets
ECM: extracellular matrix
T3 RNAP: T3 RNA polymerase
